# Antioxidant and Anti-Inflammatory Effects of Exercise in Diabetic Patients

**DOI:** 10.1155/2012/941868

**Published:** 2011-10-11

**Authors:** Saeid Golbidi, Mohammad Badran, Ismail Laher

**Affiliations:** Department of Pharmacology and Therapeutics, Faculty of Medicine, University of British Columbia, Vancouver, BC, Canada V6T 1Z3

## Abstract

Diabetes is a chronic metabolic disease which is characterized by absolute or relative deficiencies in insulin secretion and/or insulin action. The key roles of oxidative stress and inflammation in the progression of vascular complications of this disease are well recognized. Accumulating epidemiologic evidence confirms that physical inactivity is an independent risk factor for insulin resistance and type II diabetes. This paper briefly reviews the pathophysiological pathways associated with oxidative stress and inflammation in diabetes mellitus and then discusses the impact of exercise on these systems. In this regard, we discuss exercise induced activation of cellular antioxidant systems through “nuclear factor erythroid 2-related factor.” We also discuss anti-inflammatory myokines, which are produced and released by contracting muscle fibers. Antiapoptotic, anti-inflammatory and chaperon effects of exercise-induced heat shock proteins are also reviewed.

## 1. Introduction

Diabetes is a chronic metabolic disorder that continues to be a major worldwide epidemic. The prevalence of diabetes has been growing rapidly from 135 million in 1995 to an estimated 380 million in 2025 [1]. This also has consequences on the management of diabetes related complications such as cardiovascular disease, nephropathy, retinopathy, and amputations. Physical inactivity and obesity are increasingly recognized as modifiable behavioral risk factors for a wide range of chronic diseases including diabetes mellitus. The advocacy of exercise as an interventional strategy against obesity, and related metabolic diseases gains added importance from the realization that restriction of calories without exercise can lower resting metabolic rate and prevent weight loss [2]. Indeed, several studies demonstrate that physical activity and exercise alone have multiple metabolic benefits such as improved insulin sensitivity, reduced glycated hemoglobin (HbA1c), and increased peak oxygen consumption [3, 4]. 

Physical activity and exercise are defined somewhat differently. Physical activity refers to “bodily movement produced by the contraction of skeletal muscle that requires energy expenditure in excess of resting energy expenditure.” It can include a broad range of occupational, leisure, and daily activities [3]. Exercise is defined as “a subset of physical activity which are planned, structured and performed repetitively to improve or maintain one or more components of physical fitness” [5]. Exercise is classified by the type, intensity, and duration of activity. Endurance exercise reflects prolonged and continuous periods of contractile activity (high repetition) against low resistance whereas resistance exercise (strength training) involves short periods of contractile activity (low repetition) against a high opposing resistance. On the other hand, sprint exercise occurs during short periods of maximal (intense) repetitive contractile activity where there is a short period of exercise against a low resistance, such as running a 100 m sprint race. However, sprint training can also be performed against high resistance, which results in a combination of resistance and endurance modalities—for example, running with added weights. The terms physical activity and exercise will be used interchangeably in this paper. 

Although there are clear benefits of exercise in diabetic patients, a detailed understanding of the molecular basis underlying these improvements remains incomplete. An increased understanding of the molecular basis for exercise-induced metabolic effects is important in developing optimal exercise interventions for primary and secondary prophylaxis.

## 2. Diabetes and Oxidative Stress

A number of complications arise as a consequence of macro- and microvascular complications that result from diabetes; these deficits have a central role in the tissue-damaging effects of chronic hyperglycemia [6]. Since endothelial cells (as well as renal mesangial and Schwann cells) are unable to limit glucose transport as well as other cells do, they are more vulnerable to the toxic effects of hyperglycemia. In fact, from a cardiovascular medicine perspective, diabetes can also be classified as a cardiovascular disease [7]. Several studies have shown that diabetes mellitus (types I and II) is accompanied by increased formation of free radicals and decreased antioxidant capacity, leading to oxidative damage of cell components [8]. There are multiple sources of reactive oxygen species (ROSs) production in diabetes including those of mitochondrial and nonmitochondrial origins; ROS accelerates the four important molecular mechanisms involved in hyperglycemia-induced oxidative tissue damage. These four pathways are activation of protein kinase C (PKC), increased hexosamine pathway flux, increased advanced glycation end product (AGE), and increased polyol pathway flux [9].

**Figure 1:**
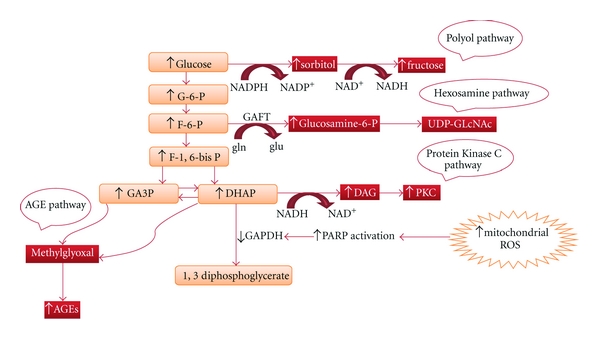
Mitochondrial ROS overproduction accelerates four hyperglycemia-induced tissue damage pathways. Dihydroxyacetone phosphate (DHAP), glutamate (glu), glutamine (gln), glutamine fructose-6-phosphate amidotransferase (GAFT), glyceraldehyde-3-phosphate dehydrogenase (GAPDH), poly (ADP-ribose) polymerase (PARP), and uridine diphosphate-N-acetylglucosamine (UDP-GLcNAc).

 Almost ten years have elapsed since Brownlee's concept of the central role of mitochondrial superoxide production in the pathogenesis of diabetic complications [10]. Briefly, he stated that increased intracellular glucose leads to an abundance of electron donors generated during the Kreb's cycle, so driving the inner mitochondrial membrane potential upward—a state that is associated with mitochondrial dysfunction and increased ROS production. This superoxide production overwhelms the capacity of MnSOD to dismutase superoxide to H_2_O_2_. These reactive oxygen and nitrogen species trigger DNA single-strand breakage to induce a rapid activation of poly (ADP-ribose) polymerase (PARP), which in turn reduces the activity of glyceraldehyde-3-phosphate dehydrogenase (GAPDH) to increase all the glycolytic intermediates that are upstream of GAPDH (Figure 1). Increased amounts of glyceraldehyde-3 phosphate directly activate two of the four aforementioned pathways, it activates AGE and PKC pathways because methylglyoxal, which is the major intracellular AGE precursor, and diacylglycerol, which activates PKC, are formed from glyceraldehyde-3 phosphate. Another upstream metabolite, fructose-6 phosphate, and also glucose, enter the hexosamine and polyol pathways, respectively. An increase in the hexosamine pathway leads to a greater production of UDP (uridine diphosphate) N-acetylglucosamine, which often results in pathologic changes in gene expression such as increased expression of transforming growth factor *β*1 and plasminogen activator inhibitor-1. Increases in the polyol pathway leads to a consumption of NADPH, a cofactor that is required for the regeneration of reduced glutathione. Activated PKC has a number of effects on gene expression such as decreased expression of eNOS and increased expressions of endothelin, vascular endothelial growth factor, plasminogen activator inhibitor-1, transforming growth factor-*β*, NAD(P)H oxidases, and nuclear factor *κ*B (NF-*κ*B), these in turn activate many proinflammatory genes in the vasculature. The activation of the AGE pathway can damage cells by three mechanisms: first, these compounds modify intracellular proteins, especially those involved in gene transcription regulation; second, these compound can diffuse to the extracellular space and modify extracellular proteins such as laminin and fibronectin to disturb signaling between the matrix and the cells; a finally, these compounds modify blood proteins such as albumin, causing them to bind to AGE receptors on macrophages/mesangial cells and increase the production of growth factors and proinflammatory cytokines [11]. The production of ROS is reduced by using either an uncoupler of oxidative phosphorylation or by the overexpression of either uncoupling protein-1 or MnSOD, such that normalizing the levels of mitochondrial ROS with any of these agents will prevent glucose-induced activation of protein kinase C, formation of advanced glycation end products, sorbitol accumulation and NF-*κ*B activation [12]. These findings support the feasibility of targeting the triggering role of mitochondrial superoxide production in hyperglycemia-induced tissue damage.

Nonmitochondrial sources of ROS include: NAD(P)H oxidase, xanthine oxidase, uncoupled eNOS, lipoxygenase, cyclooxygenase, cytochrome P450 enzymes, and other hemoproteins [27]. The structure and function of NAD(P)H oxidase was first described in neutrophils, where its superoxide production causes bacterial destruction [28]. Later work confirmed NAD(P)H oxidase production of ROS in vascular smooth muscle [29], endothelial [30] and mesangial cells [31], platelets [32], and other cell types [33, 34]. Activation of NAD(P)H oxidase in phagocytic cells is very fast (seconds) and a large burst of superoxide is produced, while activation in nonphagocytic cells takes longer (minutes to hours) and superoxide is produced continually at lower rates [29, 35]. Common stimulators of vascular NAD(P)H are angiotensin II [36], thrombin [37], platelet-derived growth factor [38], and tumor necrosis factor-*α* [39]. Inhibition of NADPH oxidase-dependent production of ROS in diabetes by a variety of PKC inhibitors suggests a regulatory role of PKC in hyperglycemia induced NADPH oxidase activity [40]. In keeping with this, PKC inhibitors decrease the expression of NADPH oxidase in high glucose-treated endothelial cells [30]. Superoxide production measured in arteries and veins from diabetic and nondiabetic patients undergoing coronary artery bypass surgery confirm an enhanced NAD(P)H oxidase-mediated production of superoxide anions in diabetics, an effect that is abrogated by chelerythrine, an inhibitor of PKC [41]. 

Xanthine oxidase and xanthine dehydrogenase are collectively referred to as xanthine oxidoreductase. While both these enzymes catalyze the conversion of hypoxanthine to xanthine and then to uric acid, xanthine oxidase reduces oxygen as an electron acceptor while xanthine dehydrogenase can reduce either oxygen or NAD+ [42]. Hydroxyl radicals, hydrogen peroxide, and superoxide are byproducts of xanthine oxidase. Even though there is some controversy about the presence of xanthine oxidase in normal endothelial cells [43, 44], it has been identified as a source of oxidative stress in the pathogenesis of atherosclerosis [45], ischemia-reperfusion [46], and diabetes mellitus [47, 48]. 

Nitric oxide is produced by inducible and constitutive nitric oxide synthases (NOSs), enzyme systems that incorporate oxygen into L-arginine. Constitutive eNOS contains reductase and oxygenase domains that are connected by a calmodulin-binding region and requires five cofactor groups (flavin adenine dinucleotide (FAD), flavin mononucleotide (FMN), heme, BH_4_, and Ca^++^-calmodulin) for activation. If eNOS lacks its substrate L-arginine or one of its cofactors, the enzyme will produce superoxide instead of nitric oxide and this is referred to as the uncoupled state of NOS. Furthermore, NO can react with superoxide to form peroxynitrite which in turn oxidizes BH_4_ and causes further uncoupling of NO formation [27].

Lipoxygenases are responsible for the conversion of arachidonic acid to leukotrienes and hydroxyeicosatetraenoic acids (HETEs) and lipoxins. They are classified according to their ability to insert molecular oxygen at the corresponding carbon position of arachidonic acid to 5-, 8-, 12-, or 15-lipoxygenase [49]. Reactive radicals, which are normally enzyme bound, are produced during the enzymatic reactions, but in some instances can also be released and attached to surrounding molecules. Lipoxygenase products, especially 12(S)-HETE and 15(S)-HETE, are involved in the pathogenesis of several diseases including diabetes where they have proatherogenic effects and mediate the actions of growth factors and proinflammatory cytokines [50, 51].

Cyclooxygenase (COX) enzymes catalyze the synthesis of various prostaglandins. The constitutive isoform COX1 is important under normal physiological conditions, while the inducible isoform COX2 is poorly expressed normally but increased dramatically during the inflammatory processes. These isoforms work in a coordinated fashion to help the body cope with diverse conditions. For example, COX2 expression is induced by proinflammatory cytokines through NADPH oxidase stimulation and ROS production. Elevated levels of glucose induce endothelium-derived vasoconstrictor prostanoids [52], suggesting a role for COX2 in diabetic vasculopathies. In addition, there is a significant correlation between plasma levels of hemoglobin A1_C_ and ligands of AGE receptors [53]. Further evidence supporting a role for oxidative stress in the induction of COX expression is that the expression of COX enzymes is normalized by glycemic control [53], and also by inhibition of oxidative phosphorylation, protein kinase C, NF*κ*B [54], or by mutation of the NF*κ*B binding elements at the COX2 promoter site [55].

The cytochrome P450 monooxygenases are a large category of enzymes involved in the metabolism and detoxification of endogenous and exogenous materials. Dioxygen compounds, which decompose and release superoxide and hydrogen peroxide, are byproducts of this process [56, 57]. Diabetes affects these different isoforms of the cytochrome P450 system; for example, there is an increased expression of CYP2E1 in type 1 and 2 diabetic [58, 59] and ob/ob mice [60], and also in STZ-induced diabetic rats [61]. The upregulation of hepatic CYP4A10 and CYP4A14 isoforms in ob/ob mice is thought to alleviate diabetes-induced hyperlipidemia since these enzymes are involved in fatty acid metabolism [62].

## 3. Exercise and Antioxidant Capacity

Cells have evolved highly complex enzymatic and nonenzymatic antioxidant systems which work synergistically, and in combination with each other, to protect the body against free radical-induced damage. The most efficient enzymatic antioxidants involve glutathione peroxidase, catalase, superoxide dismutase, heme oxygenase-1 (HO-1), NAD(P)H quinone oxidoreductase-1 (NQO-1), and thioredoxin [63]. Non-enzymatic antioxidants include vitamins E and C, thiol antioxidants (glutathione, thioredoxin) [64]. These antioxidants are capable of combining with reactive oxidants to produce other less reactive species. SOD promotes the dismutation of the superoxide radical to form hydrogen peroxide (H_2_O_2_) and oxygen. Glutathione peroxidase (GPx) uses reduced glutathione (GSH) as a reducing equivalent to reduce H_2_O_2_ to form oxidized glutathione and water. Catalase converts H_2_O_2_ to water and oxygen. Further, GSH can remove selected oxygen radicals directly and assist in the recycling of vitamins C and E. The newly identified peroxiredoxin family is also a group of peroxidases that catalyze the reduction of H_2_O_2_ and so far at least six isoforms have been identified in mammalian cells [65]. Among them, peroxiredoxin III is synthesized with a mitochondrial targeting sequence (as is MnSOD) so that when it is transferred to mitochondria, its targeting residues are cleaved during maturation. Some studies suggest that peroxiredoxin III is a critical regulator of mitochondrial H_2_O_2_ concentrations, which promotes apoptosis in cooperation with other mediators of apoptotic signaling [66]. The specific localization of peroxiredoxin III within the mitochondria is thought to provide a primary line of defense against H_2_O_2_ produced by the mitochondrial respiratory chain [67].

Exercise training results in an upregulation of antioxidant defense mechanisms in various tissues, presumably due to increased levels of oxidative stress that occurs during exercise. Low/moderate amounts of ROS produced during regular skeletal muscle work are a part of “hormesis”, which describes the generally favorable biological responses to low exposures to toxins and other stressors. A pollutant or toxin showing hormesis has opposite effects in small versus large doses. Hormesis is characterized by stimulation at low doses and inhibition at higher doses, resulting in an inverted U-shaped dose response effect [68]. For example, exercise-induced increased production of ROS can be beneficial by evoking specific adaptations, such as increased antioxidant/oxidative damage repairing enzyme activity, increased resistance to oxidative stress and lower levels of oxidative damage. On the other hand, excessive production of ROS is usually associated with detrimental effects. 

Boosting of intrinsic antioxidant potential and reduction in lipid peroxidation occurs in healthy elderly men after habitual physical activity [69]. Physiological levels of shear stress increases the expression of Cu/Zn SOD in human aortic endothelial cells [70], while endurance training mainly induces Mn-SOD expression [71]. In our experiments with db/db mice, we observed a specific down-regulation of aortic Mn-SOD following diabetes. Low-intensity exercise increased Cu/Zn-SOD protein production, whereas moderate intensity exercise increased Mn-SOD [72]. Additional work is needed to clarify the importance and physiological roles of this preferential upregulation in SODs by exercise in diabetes. 

**Figure 2:**
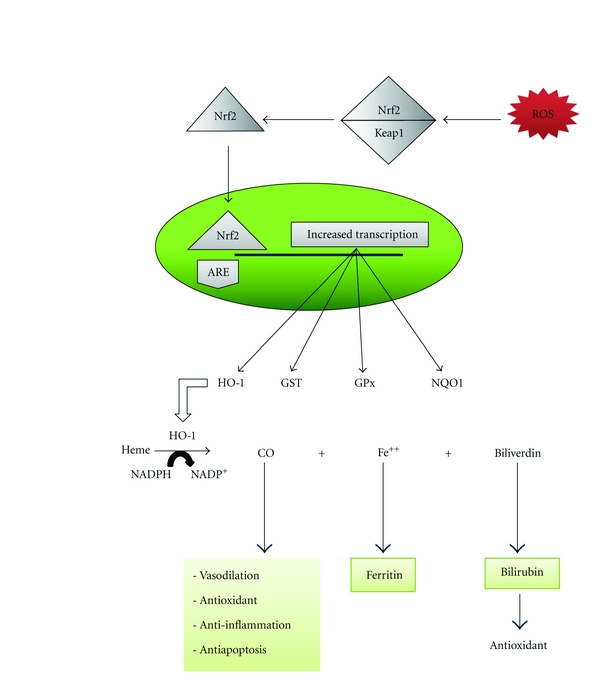
Exercise-induced ROS activates Nrf2, which then translocates into the nucleus to increase the expression of antioxidant enzymes. Antioxidant response element (ARE), carbon monoxide (CO), Glutathione peroxidase (GPx), Glutathione S-transferase (GST), Heme oxygenase-1 (HO-1), Kelch-like ECH-associated protein 1 (Keap1), NAD(P)H quinone oxidoreductase-1 (NQO-1), and nuclear factor erythroid 2-related factor 2 (Nrf2).

A critical role has recently been described for a transcription factor “nuclear factor erythroid 2-related fatcor 2 (Nrf2)” against oxidative stress in health and during diabetes. Normally, Nrf2 is located in the cytoplasm and kept dormant by a cytoplasmic repressor named Kelch-like ECH-associated protein 1 (Keap1). A variety of activators, including oxidative free radicals, release and translocate Nrf2 into the nucleus where it regulates the expression of antioxidant enzymes such as NQO-1, glutathione s-transferase, glutathione peroxidase, and HO-1 [63] (Figure 2). Diminished Nrf2 activity contributes to increased oxidative stress and mitochondrial dysfunction in the vasculature leading to endothelial dysfunction, insulin resistance, and abnormal angiogenesis as observed in diabetics [73]. HO-1, which is mainly induced through the Nrf2-keap1 signaling pathway (also known as heat shock protein 32), is the inducible isoform of heme oxygenase that catalyzes the NADPH-dependent decomposition of heme to carbon monoxide (CO), ferrous iron, and biliverdin [74]. Three isoforms of HO have been identified: both HO-2 and HO-3 are 33-kDa isoforms that are expressed constitutively [75]. The important role of HO-1 in the antioxidant defense system arises from an induction of ferritin synthesis that diminishes the cellular pool of free iron [76] and also from the enhancement of bilirubin levels, which are potent antioxidants [77]. Carbon monoxide activates soluble guanylate cyclase, a key enzyme in cell signaling that leads to vasodilation, relaxation of smooth muscle, and thrombocyte disaggregation. Carbon monoxide also affects cellular metabolism and counteracts pro-inflammatory cytokine cascades [75]. HO-1 has been widely recognized as a sensitive and reliable marker of oxidative stress [78]. Niess et al. [79] demonstrated increased cytoplasmic expression of HO-1 in human leukocytes of endurance-trained male subjects after a half-marathon run. Additionally, they determined cytoplasmic HO-1 in a control group of untrained men at rest and showed a higher expression of HO-1 compared to the athletes. They concluded that the down regulation of the baseline expression of HO-1 in athletes reflects an adaptation mechanism to regular exercise training [79]. The direct effect of exercise on Nrf2 expression has received much less attention except for a report that exercise increases nuclear levels of Nrf2 in the proximal renal tubules of old rats [80].

The effects of exercise on myocardial antioxidant enzyme activities have been widely investigated. It is generally believed that even short-term endurance exercise training results in rapid increases in myocardial Mn-SOD activity, which greatly impacts ischemic/reperfusion injury [81–83]. Exercise also increases glutathione peroxidase activity in the liver, kidney, and heart [84] as well as in skeletal muscle [85]. Exercise, and hence changes in fluid shear stress, activates vascular NADPH oxidase and P22^phox^ expression [86]. It is likely that p22^phox^ affects NADPH oxidase in response to shear stress, which may in turn regulate the amount of vascular antioxidant enzyme gene expression levels [87]. 

## 4. Diabetes, Inflammation, and Anti-Inflammatory Effect of Exercise

Inflammation has a prominent role in the pathogenesis of several cardiovascular diseases. Atherosclerosis is an inflammatory disease that is mediated by monocyte derived macrophages which accumulate in arterial plaques and become activated to release cytokines that cause tissue damage [88]. Atherosclerotic plaques in type II diabetic patients have increased inflammatory properties and worse cardiovascular outcomes than plaques observed in non-diabetic subjects [89]. We reported that systemic inflammation precedes either hyperglycemia or oxidative stress in db/db mice [90]. As evidence accumulates favoring the role of inflammation during the different phases of atherosclerosis, it is likely that markers of inflammation such as high-sensitivity C-reactive protein (hs-CRP) may be increasingly used to provide additional insights on the biological status of atherosclerotic lesions. Several studies have shown that CRP and proinflammatory cytokines, including interleukin-6 (IL-6) and tumor necrosis-*α* (TNF-*α*), are elevated in type II diabetic patients [89, 91]. CRP is considered to be an independent predictor of cardiovascular events and of the outcome of acute coronary syndromes [92]. Diabetic patients can be grouped as being at low, intermediate, and high risk for cardiovascular disease based on their levels of hs-CRP [93]. Besides its role as a marker of systemic inflammation and a predictor of cardiovascular risk, CRP and other inflammatory cytokines also directly trigger vascular dysfunction [94], possibly via altering calcium channel expression and activity [95], upregulation of Rho-kinase expression and function [96], increasing the production of ROS [97], and/or enhancing cyclooxygenase expression [98]. In turn, cyclooxygenase enzymes cause vascular hypercontractility by increasing the synthesis of constrictor prostanoid(s) [99, 100] and excessive formation of ROS [101]. Cyclooxygenase inhibitors alleviate the augmented contractile responses in several animal models of diabetes [102–106]. These findings may partially explain the inconsistent and mostly disappointing results with antioxidant use in diabetic patients [64], since inflammation rather than oxidative stress may be the principle contributor to diabetic vascular dysfunction. In agreement with this concept is the finding that endothelial function improves in type 2 diabetic patients treated with rosiglitazone, an agent that reduces inflammation but not oxidative stress [107].

Exercise produces a short-term inflammatory response that is accompanied by leukocytosis, increases in oxidative stress, and plasma levels of CRP. This pro-inflammatory response is followed by a long term anti-inflammatory effect [108]. Regular exercise reduces CRP, IL-6, and TNF-*α* levels and also increases anti-inflammatory substances such as IL-4 and IL-10 [109, 110]. In healthy young adults, a 12-week, high-intensity aerobic training program down regulates cytokine release from monocytes [110]. In fact, even leisure time physical activity (e.g., walking, jogging, or running, etc.) reduces hs-CRP concentration in a graded manner [111].  Table 1 summarizes the findings of clinical studies on the effects of exercise on anti-inflammatory and antioxidant markers in diabetic patients.

## 5. Myokines as Anti-Inflammatory Agents

Pedersen and colleagues [112–116] suggest that just as adipose tissue is recognized as an endocrine organ, skeletal muscle should also be considered as an endocrine tissue. The term “myokines” was later coined for cytokines and other peptides that are produced, expressed, and released by muscle fibers. The list of myokines includes IL-6, IL-8, IL-15, brain-derived neurotrophic factor, leukemia inhibitory factor plus fibroblast growth factor-21, and follistatin like-1 [113]. They are released from working muscles into the circulation where they exert their effects on other organs in a hormone-like fashion. Myokines are thought to mediate the beneficial effects of exercise and may also have a role in the protection against diseases associated with low-grade inflammation such as atherosclerosis, type II diabetes, or the metabolic syndrome.

IL-6 is the first cytokine released into the circulation during exercise and its levels increase in an exponential fashion in response to exercise (99). IL-6 mRNA is upregulated in contracting skeletal muscle [117] and the transcriptional rate of the IL-6 gene is also markedly enhanced by exercise [118]. IL-6 acts as both a proinflammatory and anti-inflammatory cytokine. When secreted by T cells and macrophages, IL-6 stimulates immune responses and boosts inflammatory reactions, while muscle-produced IL-6 exerts anti-inflammatory effects through its inhibitory effects on TNF-*α* and IL-1*β*, and activation of IL-1ra and IL-10 [115]. Exercise-induced increases in plasma IL-6 correlate with the muscle mass involved in exercise activity and also with the mode, duration, and especially intensity of exercise [119]. Exercise also confers protection against TNF-induced insulin resistance [120]. In addition, Starkie et al. reported that infusion of recombinant human IL-6 (rhIL-6) into human subjects simulated the exercise induced IL-6 response in the prevention of endotoxin-induced increase in plasma TNF-*α* [121]. Exercise can also suppress TNF-*α* production by an IL-6 independent pathway, as demonstrated by Keller et al. who reported only modest decreases in plasma TNF-*α* after exercise in IL-6 knockout mice [122]. Exercise induced increases in epinephrine levels can also blunt the TNF-*α* response [123]. In addition, Petersen et al. showed that IL-6 enhances lipid turnover and stimulates lipolysis as well as fat oxidation via activation of AMP-activated protein kinase [124]. Consistent with this, Wallenius et al. demonstrated that IL-6 deficient mice (IL6−/−) develop mature onset obesity and have disturbed carbohydrate and lipid metabolism that is partly reversed by IL-6 replacement. Other data indicates that centrally acting IL-6 exerts an antiobesity effect in rodents [125]. The lipolytic effect of IL-6 on fat metabolism was confirmed in two clinical studies of healthy and diabetic subjects [124, 126]. Visceral fat is potentially a cause of low-grade systemic inflammation, which in turn leads to insulin resistance, type II diabetes, and atherosclerosis [113]. During exercise, IL-6 also increases hepatic glucose production. Glucose ingestion during exercise reduces IL-6 production by muscles, suggesting that IL-6 is released due to of the reduction in glycogen levels during endurance exercise and the consequent adrenergic stimulation of IL-6 gene transcription via protein kinase A activation [127].

## 6. Heat Shock Proteins

There is widespread clinical interest in the role of heat shock proteins (HSPs) in a number of human diseases, including diabetes. The heat shock response is a common cellular reaction to external stimuli such as ischemia [128], hypoxia [129], acidosis [130], oxidative stress [131], protein degradation [132], increased intracellular calcium [133], and energy depletion [134]. Therefore, the terms “stress proteins” and “cellular stress response” reflect the array of stressors known to initiate HSP expression [135]. Heat shock proteins are grouped into six major families based on their molecular weight and related functions, that is, 110 kDa HSPs, 90 kDa HSPs, 70 kDa HSPs, 60 kDa HSPs, 40 kDa HSPs, and small HSPs such as HSP27, *α*B-crystallin, and ubiquitin. Some HSPs are constitutively expressed in cells (e.g., HSP90, HSP70), while other HSPs are rapidly and highly inducible in response to stress (e.g., HSP70, HSP27) [136, 137]. Several important cytoprotective functions have been attributed to these proteins including (a) folding of proteins in various intracellular compartments, (b) maintenance of structural proteins, (c) refolding of misfolded proteins, (d) translocation of proteins across membranes into various cellular compartments, (e) prevention of protein aggregation, (f) degradation of unstable proteins, and (g) apoptosis [137]. An increased content of HSPs promotes cellular recovery by binding with misfolded and unfolded proteins and promoting the refolding of these proteins when cellular conditions improve [138]. Thus, an important role for the increased expression of HSPs is to function as molecular chaperones, having the features of a feedback system that reacts to increased misfolded proteins by elevating the synthesis of the chaperones that helps in the refolding process [139]. HSPs also have roles as antioxidants and in the inhibition of apoptosis and inflammation [140]. Levels of HSP72 mRNA in skeletal muscle decrease in patients with type II diabetes and this may be related with insulin resistance [141–143]. Studies in animals show that heat shock therapy, regardless of the way used to achieve HSP elevation (transgenic overexpression or pharmacologic means to overexpress HSP72 protein expression), protects against diet- or obesity-induced hyperglycemia, hyperinsulinemia, glucose intolerance, and insulin resistance [144]. Suggested mechanisms for the reduction of HSPs in diabetes include the following: insulin has regulatory roles in both the initiation and elongation phases of translation by altering the phosphorylation of eukaryotic translation initiation factors and eukaryotic elongation factors, therefore its impaired or deficient secretion in diabetes may be one explanation for attenuated (stress) protein synthesis [142]. Suppression of heat shock transcription factor-1 (HSF-1) via upregulation of glycogen synthase kinase, an enzyme originally described as a regulator of glycogen metabolism [143]. The reduced HSF-1 and HSP levels in diabetes could result from decreased membrane fluidity and compromised membrane integrity. Vigh et al. considered how the physical state of the membrane could affect gene expression and, hence, responses to stress: they proposed that alterations in plasma membrane microdomains are well suited for sensing stress and retailoring the expression of the various classes of HSPs [145]. Some pathological conditions, including diabetes, which are associated with membrane defects, can be improved with insulin therapy [146].

Prolonged exercise of sufficiently high intensity creates physiological stresses and disturbs cellular homeostasis, leading to induction of cellular adaptation mechanisms. There is an increased HSP70 mRNA concentration after just 4 min of a single bout of exercise at anaerobic threshold in human subjects [44]. In another study in humans, Walsh et al. [147] demonstrated increased HSP72 mRNA expression in skeletal muscles 2 hours after exercise and increased serum HSP72 protein. The increase in serum HSP72 preceded any increase in HSP72 gene or protein expression in contracting muscle, suggesting that HSP72 was released from other tissues or organs, suggesting a systemic role for the protein. Although increased muscular HSP transcription occurs during exercise, immediately after exercise or several hours after it, a brisk increase in protein content is only detectable after 1-2 days following the exercise stress [139].

## 7. Activation of HSPs in Skeletal Muscles

The redox-signaling pathway is the main mechanism for induction of the stress response during endurance exercise. Thus, Fischer et al. [148] reported that a combination of vitamin C and E for 28 days prevented increases in muscle HSP27 mRNA expression as well as circulating levels of HSP72 protein. This finding is supported by data from other investigators who also reported that the increased HSP70 content in human skeletal muscle following exercise was abolished by antioxidant therapy [149, 150]. Antioxidant therapy scavenges exercise induced ROS, thereby abolishing transcriptional activity of the *HSP* gene [139]. It is also likely that antioxidants increase baseline muscle HSP70 content, so explaining the diminished response during stress [149, 150]. In addition to aerobic exercise protocols, several studies have used resistance exercise and downhill running protocols to study HSP expression in human muscle [151, 152]. These protocols are considered more damaging since they induce overt structural and mechanical damage to the muscles. Increases in HSP27, HSP70, and *α*B-crystallin have also been reported in these studies. It is difficult to compare the results of these studies with those using nondamaging exercise protocols, since data from damaging exercise protocols are complicated by inflammatory responses; for example, phagocyte cells, which infiltrate 2-3 days after damaging exercise, contain relatively high levels of HSP, and neutrophils are also sources of superoxide that can induce HSP production.

## 8. Antiapoptotic and Anti-Inflammatory Effects of HSPs

The HSP70 family is the most abundant HSP and mostly includes the constitutive cytosolic HSP73 and the stress-induced cytosolic HSP72. In addition to their chaperone functions, some beneficial effects are attributed to their antiapoptotic and anti-inflammatory effects. Apoptosis, the process of programmed cell death that occurs in multicellular organisms, can originate either extracellularly (by activation of specific death receptors) or intracellularly [153]. Mitochondria play an important role in the regulation of apoptosis. They contain several proapoptotic proteins such as cytochrome C, apoptosis inducing factor (AIF), and second mitochondria-derived activator of caspases (SMACs). These factors are released from the mitochondria following the formation of a pore in the mitochondrial membrane called the permeability transition pore, or PTP. These pores are thought to result from apoptotic signals such as cell stress, free radical damage, or growth factor deprivation [154]. Once cytochrome C is released, it binds with apoptotic protease activating factor-1 (Apaf-1) and ATP, which then bind to procaspase-9 to create a protein complex known as an apoptosome. The apoptosome cleaves the procaspase to its active form of caspase-9, which in turn activates the effector caspase-3. Upon release of SMAC, it binds to inhibitor of apoptosis proteins (IAPs) and deactivates them, preventing the IAPs from arresting the apoptotic process and therefore allowing apoptosis to continue. IAPs normally suppress the activity of caspases, which carry out the degradation of the cell. When AIF is released from mitochondria, translocates to the nucleus, where apoptosis occurs in the absence of caspase activation (caspase-independent pathways) [155]. Mitochondrial release of cytochrome C and AIF can be antagonized by bcl-2. The bcl-2 proteins are a family of proteins involved in the response to apoptosis. Some of these proteins (such as bcl-2 and bcl-XL) are anti-apoptotic, while others (such as Bad, Bax, or Bid) are pro-apoptotic. The sensitivity of cells to apoptotic stimuli depends on the balance of pro- and antiapoptotic bcl-2 proteins [156].

HSP70 affects the apoptosis death cascade at different levels. It can inhibit caspase activation by interfering with Apaf-1 and prevent the recruitment of procaspase-9 to the apoptosome [157, 158]. HSP70 also increases Bcl-2 expression and inhibits cytochrome C release; it also binds and sequesters AIF [159, 160]. Overexpression of HSP70 in lymphoid tumor cell lines appears to inhibit apoptosis by blocking caspase activation and activity [161]. The anti-apoptotic effects of HSP70 have been studied in brain tissue, where mice overexpressing HSP70 had decreased infarct sizes, improved neurological deficits, fewer apoptotic cells, and reduced DNA laddering after middle cerebral artery occlusion [162, 163].

As an anti-inflammatory molecule, HSP70 decreases the release of inflammatory mediators in different models of inflammation [164–166]. It has been suggested that HSP70 interacts with NF-*κ*B to exert this anti-inflammatory effect. NF-*κ*B is a ubiquitous transcription factor that plays an essential role in inflammatory responses to a variety of signals, immune function, endothelial cell activation, and the control of cell growth [167–169]. NF-*κ*B is normally located in the cytoplasm in an inactive form by virtue of binding to a family of inhibitor of NF-*κ*B (I*κ*B) proteins. Upon cell stimulation by a wide variety of stimuli, signal responsive IKK*α* and –*β* (TNF-a-inducible IkB kinase complex also known as IKK-1 and IKK-2) are activated, which results in the phosphorylation of I*κ*B and its proteasomal degradation. I*κ*B degradation liberates NF-*κ*B, allowing it to translocate to the nucleus and induce gene expression. Induction of HSP72 in vitro (by heat shock or HSP72 overexpression) regulates the expression of inflammatory genes such as TNF-*α*, IL-1, IL-12, IL-10, and IL-18 [165, 166, 170–172]. Ran et al. [173] showed that HSP70 can, in fact, directly interact with IKK-*γ* and prevent I*κ*B phosphorylation. Chronic activation of IKK occurs in diabetic patients and it has been shown that the reduced IKK activity of the NF-*κ*B pathway prevents the development of insulin resistance in vitro and in vivo [174, 175]. High-fat, high-carbohydrate meals cause a greater and more prolonged oxidative stress and NF-*κ*B activation in obese subjects [176]. Therefore, it can be concluded that IKK and NF-*κ*B activation are among the most important negative regulators in the development of type II diabetes that are opposed by HSPs.

Another mechanism for the anti-inflammatory effects of HSP72 involves inhibition of high-mobility group box 1 (HMGB1) release. HMGB1 is a pro-inflammatory nuclear protein that mediates responses to infection, injury, and inflammation [177]. It is secreted by activated macrophages in response to exogenous and endogenous inflammatory stimuli (such as endotoxin, TNF-*α*, IL-1, IFN-*γ*, and hydrogen peroxide) and is released passively by necrotic and damaged cells [178]. Upon its release, HMGB1 binds to cell surface receptors including the receptors for AGEs, toll-like receptor 2 (TLR2), and TLR4. AGEs receptors are expressed on endothelial and smooth muscle cells, monocytes/macrophages, neurons, and in several malignant and transformed cells [179]. Fiuza et al. [180] showed that HMGB1 stimulates human endothelial cells to increase the expression of intercellular and vascular adhesion molecules (ICAM-1 and VCAM-1), AGEs receptors as well as proinflammatory cytokine (TNF-*α*) and chemokines (IL-8 and monocyte chemotactic protein-1). This proinflammatory phenotype is mediated in part by early TNF-*α* secretion and involves the activation of stress mitogen activated protein kinase (MAPK) pathways and the transcription factor NF-*κ*B [180]. MAPKs and the NF-*κ*B/I*κ*B pathway play important roles in inflammation because of the rapidity and extent of activation of transcription of NF-*κ*B [181]. High circulating levels of HMGB1 occur in type I and type II diabetic patients [182, 183]. In an animal model, hyperglycemia induced by infusion of glucose also elevates serum levels of HMGB1 [184]. Other important actions of HSP in the inflammatory response include HSPs reduce LPS-induced HMGB1 release from macrophage cultures [185]; HSP72 negatively regulates oxidative stress-induced HMGB1 cytoplasmic translocation and release [186] and HSP72 overexpression inhibits HMGB1-induced cytokine (TNF-*α*, IL-1*β*) expression and release via inhibition of the MAPKs and NF-*κ*B pathways [187].

## 9. Summary

The key roles of inflammation and oxidative stress in the pathogenesis and progression of diabetes are well accepted and their biomarkers are being increasingly used in the management and risk assessment of diabetic patients. There are multiple sources of ROS production in diabetes including those of mitochondrial and non-mitochondrial origins; increased production of ROS and a concomitant decline of antioxidant defense mechanisms leads to damage of cellular organelles and enzymes and development of insulin resistance. Emerging evidence suggests that exercise activates the expression of cellular anti-oxidant systems and there is evidence to suggest that Nrf2 plays a critical role in this regard. Exercise produces a short-term pro-inflammatory response that is followed by a long-term anti-inflammatory effect. Regular exercise is associated with lower levels of CRP, IL-6 and TNF-*α* and, simultaneously, with increases in anti-inflammatory substances such as IL-4 and IL-10. The health-beneficial effects of exercise-induced myokines and heat shock protein and their proposed mechanisms are gaining increased recognition.

## Figures and Tables

**Table 1:** Summary of clinical studies on the anti-inflammatory and antioxidant effects of exercise in diabetic patients reported during the last 10 years.

Reference	Patient characteristics	Exercise duration	Measured parameters	Outcome
[13]	14 T2D versus 12 healthy subjects	18 weeks	(i) Ability of HDL subfractions to inhibit LDL oxidation in vitro. (ii) Serum PON activity. (iii) Total antioxidant status. (iv) Plasma lipid peroxidation.	(i) Exercise improved the antioxidant role of HDL and reduced plasma lipid peroxidation in diabetic subjects.

[14]	50 T2D versus 20 age matched controls	Single bout (exercise cycle ergometer test)	(i) TG, TC, LDL, oxLDL, SOD, GSH-Px, PAI.	(i) Exercise increased oxLDL and SOD in both groups; GSH-Px was increased only in diabetic patients.

[15]	13 diabetic men	3 weeks combination of high-fiber, low-fat diet plus aerobic exercise	(i) Serum lipids, glucose, insulin, 8-iso-PGF2*α*, CRP, sICAM-1, sE-selectin (ii) in vitro measurement of NO, superoxide, and H_2_O_2_ (iii) Serum-induced monocyte adhesion, ICAM-1, VCAM-1, MCP-1 in cultured endothelial cells.	(i) Reduction in TC, LDL, FSG, insulin, 8-iso-PGF2*α*, CRP, sICAM-1, sE-selectin, serum stimulated monocyte adhesion, cellular ICAM-1 and VCAM-1, superoxide and H_2_O_2_ and increase in NO production.

[16]	134 T2D divided into 3 groups (i) 43, aerobic training plus using fitness center (ii) 44 aerobic training only (iii) 16 controls	12 months	(i) Urinary 8-OHdG (ii) serum glycated albumin, TC, HDL, TG, HbA1c.	(i) Urinary 8-OHdG decrease after 12 months in the exercise groups.

[17]	16 T2D with diet restriction and 13 T2D with diet restriction and exercise	12 weeks	(i) MDA, 24 h urinary nitrate/nitrite, FMD (ii) BW, waist circumference, BP, HbA1c, glucose, insulin resistance, lipid profile.	(i) Both interventions reduced BW, waist circumference, BP, HbA1c, glucose, insulin resistance, lipids and MDA, and increased urinary nitrite/nitrate ratio (ii) No change in FMD.

[18]	(i) 77 T2D in yoga group (ii) 77 T2D in conventional exercise group (iii) 77 T2D as controls	6 months	(i) FBS, TC, TG, LDL, VLDL, HDL, MDA, POX, PLA2, SOD, catalase activity.	(i) Significant reductions in FBS, TC, VLDL, MDA (ii) SOD increased. (iii) No significant changes in PLA2 and catalase activity.

[19]	(i) 56 T2D in t' ai chi chuan (TCE) group (ii) 48 conventional exercise group (CE)	12 weeks	(i) HbA1c, lipid profile, MDA, CRP (ii) BW, BMI.	(i) BMI, serum lipids, MDA, and CRP significant improved in TCE group. HbA1c did not decrease. (ii) No improvement in BMI, lipids, and oxidative stress profiles in the CE group.

[20]	(i) 12 sedentary nondiabetes subjects (ND) (ii) 12 sedentary T2D (T2S) (iii) 9 physically active (T2DA)	Single bout of intense exercise (>85% VO_2max_)	(i) FBS, HbA1c, body fat percent, lipid profile (ii) TBARS, GSH.	(i) T2DS had higher FBS, HbA1c, and body fat percent than T2SA. (ii) T2DA had higher VO_2max_ spent more time on treadmill, lower Hb and BP compared with ND and T2DS. (iii) TBARS in T2DS were higher than T2DA. (iv) GSH was similar among groups.

[21]	11 T2D	10 weeks	(i) Muscle strength, Wmax, VO_2max_, MUOX, IMCL and IMCG, systemic inflammatory markers and primary diabetic outcome measures plus daily exogenous insulin requirements (EIRs).	(i) Muscle strength and Wmax increased. (ii) Mean arterial BP and EIR, FBG and nonesterified fatty acids declined. (iii) No changes in VO_2max_, MUOX, IMCL or IMCG, HbA1c, adiponectin, TNF-*α*, and cholesterol

[22]	20 T2D (sedentary control, A) 20 T2D (low intensity aerobic exercise, B) 20 T2D (high intensity aerobic exercise, C) 22 T2D (aerobic and resistance exercise, D)	12 months	HbA1c, FBS, TG, TC, HDL, hs-CRP, IL-1*β*, IL-4, IL-6, IL-10, TNF-*α*, IFN-*γ*, leptin, resistin, adiponectin VO_2max. _	(i) Significant decrease of hs-CRP in groups C and D. (ii) leptin, resistin, IL-6 decreased in groups C&D, while adiponectin increased. (iii) IL-1*β*, TNF-*α*, IFN-*γ* decreased in group D, whereas anti-inflammatory IL-4 & 10 levels declined.

[23]	406 T2D out of 522 participants from the Finnish Diabetes Prevention Study	1 year	CRP and IL-6 levels.	(i) Increases in fiberintake and moderate to vigorous leisure time physicalactivity (LTPA), but not total LTPA, predicted decreases inCRP and/or IL-6.

[24]	15 T2D (control) 15 T2D (10000 steps/day)	6 weeks	(i) Anthropometric measures plus, HbA1c, FBS, insulin, lipid profile, fructosamine, total radical antioxidant parameter, PAI-1, homocysteine and lipoprotein(a).	(i) HDL and resting energy expenditure increased while PAI-1 levels decreased in the active group.

[25]	25 T2D (exercise group) 25 T2D (control group)	16 weeks	(i) Anthropometric measures and insulin resistance, MMP-2, TIMP-1, lipid profile, HbA1c, fibrinogen, hsCRP, VO_2max_, VT.	(i) Systolic and mean BP, LDL, HbA1c, fibrinogen, hsCRP, MMP-9 and MMP-9 to TIMP-1 ratio decreased in exercise group while VO_2max_, VT and plasma TIMP-2 levels increased.

[26]	60 patients with IGTT: 24 in exercise training group 20 in rosiglitazone group 16 in control group	12 months	(i) Genotyping of the 174G/C IL-6 variant. (ii) CRP and IL-6 measurements.	(i) Improved peak VO_2max_, decreases in BMI, WHR, HbA1c, plasma glucose and insulin, 2-h OGTT glucose level IL-6 and hsCRP in exercise group.

Abbreviations: 8-OHdg: 8-hydroxy-2′-deoxyguanosine, BMI: body mass index, BP: blood pressure, BW: body weight, CRP: C reactive protein, FBS: fasting blood sugar, FMD: flow mediated dilatation, GSH: glutathione, GSH-Px: glutathione peroxidase, HbA1c: hemoglobin A_1c_, HDL: high density lipoproteins, ICAM-1: intracellular adhesion molecule-1, IFN: interferon, IL: interleukin, IMCG: intramyocellular glycogen, IMCL: intramyocellular lipid, LDL: low density lipoprotein, MCP-1: monocyte chemotactic protein-1, MDA: malondialdehyde, MMPs: matrix metalloproteinases, MUOX: muscle oxidative capacity, oxLDL: oxidized low density lipoproteins, PAI: plasminogen activator inhibitor, PLA2: phospholipase A2, PON: paraoxonase, POX: protein oxidation, sICAM: soluble intracellular adhesion molecule, SOD: superoxide dismutase, T2D: type II diabetes, TBARS: thiobarbituric acid substances, TC: total cholesterol, TG: triglyceride, TIMPs: tissue inhibitor of metalloproteinases, VCAM-1: vascular cell adhesion molecule-1, VLDL: very low density lipoprotein, VO_2max_: whole body peak oxygen uptake, VT: ventilatory threshold, and Wmax: maximal workload capacity.
